# Prevalence and prescribers’ knowledge of psychotropic polypharmacy in the Bono, Bono East, and Ahafo Regions, Ghana

**DOI:** 10.1016/j.heliyon.2024.e24243

**Published:** 2024-01-08

**Authors:** James Dumba, Antwi Joseph Barimah, Mohammed Mohammed Ibrahim, Solomon Saka Allotey, Semefa Alorvi, William Appertey, Luke Sopaal, Frank Acheampong, Rebecca Dorcas Commey, Yaw Boakye Nketiah, Deborah Ampofo, Bernard Opoku Amoah, Larry Agyemang

**Affiliations:** College of Health Yamfo, Ghana

**Keywords:** Prevalence, Knowledge, Psychotropic, Polypharmacy, Bono, Bono east, Ahafo regions, Prescribers, Medications, Antipsychotic

## Abstract

The use of psychotropic medications for treating simple and complex psychological and emotional problems is common and relevant among prescribers. This, therefore, come with the tendency to prescribe many medications to a patient on a single visit due to varied reasons. The study, therefore, sought to ascertain the prevalence and prescribers' knowledge of psychotropic polypharmacy. A quantitative, descriptive study was conducted using a simple random approach to select and review the prescription records (both regular and prn basis) of three hundred and nine (309) patients’ folders retrospectively within seven (7) mental health units across the study area to ascertain the prevalence of Psychotropic Polypharmacy (PP). Fifty-eight (58) prescribers were selected using probability method (simple random) to respond to the study questionnaire. Psychotropic Polypharmacy was prevalent (66.0 %) in the study area with antipsychotic polypharmacy (74.0 %) being the common form with the co-prescription of chlorpromazine (CPZ) + haloperidol (70.0 %) being frequent. This was more predominant among male patients (78.0 %) than female patients (22.0 %). Prescribers had high (82.8 %) knowledge of Psychotropic Polypharmacy and the majority (68.9 %) disagreed that the practice of Psychotropic Polypharmacy should be promoted. The continuous training of prescribers (i.e. mental health officers) on rational prescriptions would help reduce the prevalence of Psychotropic Polypharmacy.

## 1 Introduction

Psychotropics medications are classes or variety of drugs that act on the mind or are used to treat various conditions and/or disorders of the human mind. These drugs affect mood, behaviour, thoughts or perception [1].

Psychotropic medications have also been used over the period worldwide as a means of relieving or curing the signs and symptoms of mental illnesses/disorders, maintaining stability, prevention of relapses, and complications. Studies conducted have shown increasing degrees of psychotropic drug use and the concurrent use of multiple psychotropic medicines (polypharmacy) among children overall between 1 and 5years as well as in children with Autism Spectrum Disorders (ASD) [1].

According to the World Health Organization, the term polypharmacy is the administration of many drugs at the same time or the administration of an excessive (2 or more) number of drugs. However, the debate on polypharmacy is not just about the number of drugs used, but also about the efficacy, usefulness and possible harm of each drug, either individually or in combination [2].

Globally, there seems to be no consensus on the precise concept of polypharmacy, but most meanings are numerical in sense. Polypharmacy is the use of multiple drugs to treat a patient's medical condition. The basis for this concept is strictly quantitative and does not consider the clinical importance of the use of these drugs (for example, the existence of multiple diseases) or the adequacy of the suggested therapeutic regimen. The term polypharmacy indicates that more medications are used than ‘clinically suggested.’ However, the number of drugs that constitute polypharmacy has not been specified in the available literature [3].

The World Health Organization (WHO) has highlighted polypharmacy among prescribers as one of the major areas of focus in its Third Global Patient Safety Challenge, Medication without Harm [4]. Polypharmacy is known to occur when the possible advantages of multiple medicines outweigh the detrimental consequences of a patient's sheer number of medications prescribed. The incidence of polypharmacy is not simply about the number of drugs a patient uses, but also about the efficacy, usefulness, and possible harm of the drug either individually or in combination [3].

Polypharmacy is a worldwide concern in both physical and mental health facilities [5]. Globally, there appears to be a recounted high predominance of psychiatric or psychotropic polypharmacy (PP) especially in children and adolescents which ranges from 2.9 to 27 % [6].

It is said that the rate of polypharmacy is more prevalent among elderly patients and people with mental health problems, both of which are particularly at risk of adverse outcomes [4].

Polypharmacy in psychiatry is an understudied area in Africa as few results are available for use. While antipsychotic polypharmacy practices are observed with growing frequency by prescribers, few studies have defined patient behaviors and treatment behaviors associated with the long-term use of this treatment strategy [7].

This, therefore, necessitated the investigation of the prevalence and prescribers’ knowledge of Psychotropic Polypharmacy in the Bono and Ahafo Regions of Ghana.

## 2 Materials and methods

### 2.1 Study design

A quantitative study using descriptive parameters was employed to ascertain the prevalence and prescribers' views and the effects of psychotropic polypharmacy in the Bono, Bono East, and Ahafo regions of Ghana. A descriptive study deals with the collection of data meant to explain or predict the conditions or relationships that may exist, opinions of subjects as well as practices that are going on. Data was collected between October and November 2020.

### 2.2 Inclusion and exclusion criteria/indicators

Patients (their respective folder/records) who were diagnosed with psychological disorders including psychotic, mood, and anxiety disorders were included in the study. Prescription charts (secondary data) across mental health units within the 3 regions were reviewed retrospectively (data from patients' recent or last three review visits were used). This was to identify all patients diagnosed with psychotic conditions as well as to determine those who were co-prescribed with two or more antipsychotics either on regular and/or prn basis. 309 patients’ folders (both inpatients and outpatients) within the respective mental health units were reviewed randomly but systematically (documented continuous prescription) from January 1, 2019 through to March 30, 2020. Two (2) to eleven (11) or more medications prescribed to a patient within the same time of interaction with the prescriber with the use of medication(s) from the same drug category or class.

Only qualified and permanent staff (prescribers) working within these regions were randomly selected to respond to the questionnaire. Therefore, national service and rotational staff were excluded from this study. Also, mental health units that do not record a daily average OPD attendance of 10 patients were excluded from this study. Only clients or patients undergoing either pharmacotherapy alone or both pharmacotherapy and psychotherapy were used for this study.

Psychotropic polypharmacy was regarded in this study only if two or more psychotropic drugs (i.e., antipsychotics, anticholinergics, antidepressants, mood stabilizers, stimulants, etc.) are used or prescribed during the same treatment time/concurrently (i.e., same class, multi-class, adjunctive and increase polypharmacy), not complete polypharmacy.

### 2.3 Sample size and sampling method

The study utilized 309 patient folders and 58 prescribers.

To obtain the needed data for the study objectives, a random sampling method was used to select patients' folders. However, a purposive sampling method was used to select the mental health units/facilities for data collection within the Bono, Bono East, and Ahafo regions of Ghana. Sample (patients’ folders) distribution and the selection were purposefully done based on size and Outpatient Department (OPD) attendance rate (average 10 patients daily) of the selected facilities as follows; Bono regional hospital: 131, Abrefi Hospital, Techiman 44, Kintampo government hospital 28, Goaso government hospital 41, Berekum hospital 37 and Dorman Presbyterian hospital 28.

Prescribers were randomly selected proportionally at the respective Mental Health Units based on the number per facility as follows; Bono regional hospital: 15, Abrefi Hospital-Techiman: 5, Kintampo government hospital: 7, Berekum Hospital: 3, Wamfie government hospital: 4, Dormaa Presbyterian hospital: 4, Goaso government hospital: 7, Bechem government hospital: 3 and Sunyani municipal hospital:10. These 3 regions were selected because they form core and a good proportion of mental health care in the country especially for the middle belt of the country.

The random sampling method was adopted because it takes a small proportion of the entire population to represent the entire data set ensuring that each subject has an equal chance of being selected for the study.

### 2.4 Analysis of data

Data collected from the field was analyzed with the aid of SPSS (version 25) using Stata version 6.0 for entry and analysis. Results are presented in tables, graphs, and charts representing the major findings relative to the objectives of this study. A chi-square correlational test was used to determine the association between a prescribers’ knowledge level and demographic characteristics using 95 % confidence interval, a *P*-value of less than 0.05 was considered significant in this study.

### 2.5 Ethical consideration

The Committee on Human Research and Publication Ethics (CHRPE), School of Medical Sciences, KNUST accordingly approved and gave ethical clearance as the protocol for the conduct of this study which bears the reference number CHRPE/AP/325/20.

The needed ethical clearance or approval was also sought from the respective hospital or unit heads before conducting this research via the Bono and Ahafo regional health directorates. The respondents were allowed to stay off from the exercise at any point in time. The participants were assured of anonymity, confidentiality, privacy, beneficence as well as non-maleficence. Thus, they were assured that the exercise was purely academic and that it would be of importance to them and would not cause any of form harm to them. Hence, no participant was coaxed or forced into partaking in this study. They were also told not to provide any traceable personal information on the tool. All articles and other academic resources used for this study were properly referenced.

## 3 Results

As captured in Table 1 above, this study was conducted among two categories of respondents which were clients (data collected using their respective folders) and prescribers (mental health workers). Respectively, there were 309 clients folders and 58 prescribers across the study who willingly took part in this study. With 33 years as the mean age and a standard deviation of 14.6 among the clients, a lot of them were between the ages of 21–30 years. Only a few (3.6 %) were below 10yrs. More than half of the respondents (clients) were males (59.9 %) with the remaining as females.

**Table 1:** Demographic characteristics of clients.

Variable	Freq. (N = 309)	Percentage (%)
***Age groups***		
10yrs & Below	11	3.6
11–20yrs	52	16.8
21–30yrs	84	27.2
31–40yrs	77	24.9
41–50yrs	40	12.9
51–60yrs	33	10.7
61yrs & Above	12	3.9
**Mean age (STDV.)**	**33yrs (14.6)**	
***Gender***		
Male	185	59.9
Female	124	40.1
***Facility***		
Berekum Health Center	37	12.0
Dormaa MHD	28	9.1
Goaso Government Hospital	41	13.3
Kintampo Municipal Hospital	28	9.1
Regional Hospital-Sunyani	131	42.4
Techiman polyclinic	44	14.2

From Table 2, among the prescribers, the majority were within the age group of 21–30 years. The majority of the respondents were males (62.1 %) like the clients' group. About half of them (51.7 %) were singles whilst the remaining were married prescribers. More than half (62 %) of them were registered mental health nurses (s) as per their qualifications.

**Table 2:** Demographic characteristics of the respondents (prescribers).

Variable	Freq. (N = 58)	Percentage (%)
Age groups		
20–30yrs	35	60.3
31–40yrs	22	37.9
41–50yrs	1	1.7
***Gender***		
Male	36	62.1
Female	22	37.9
***Marital Status***		
Married	28	48.3
Single	30	51.7
***Qualification***		
RMN	36	62.1
CMHO	21	36.2
Others	1	1.7

### 3.1 Prevalence of psychotropic polypharmacy

The study investigated clients' folders (prescribing records) to find out how often PP occurs. This was done by identifying and categorizing clients based on the number of medications prescribed to them in each facility visited. These groupings were then used to ascertain the prevalence of PP as indicated in Fig. 2 below.

**Fig. 1:**
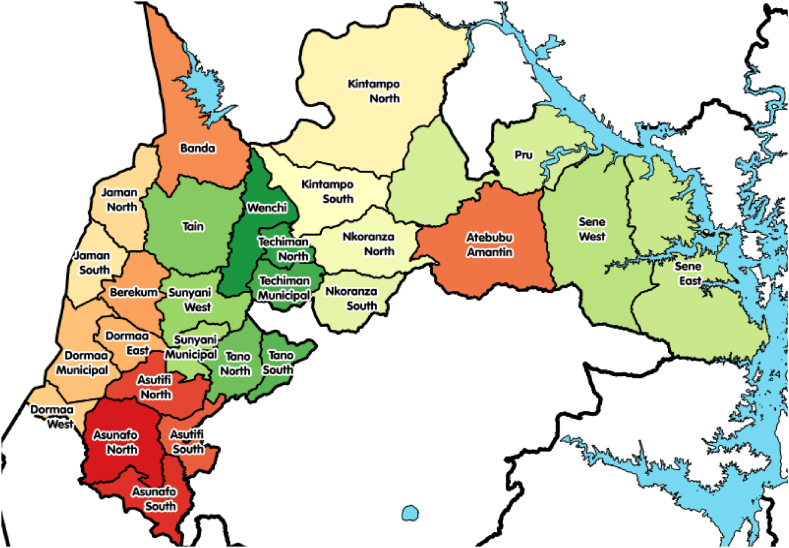
Map of bono, bono east, and ahafo regions of Ghana.

Findings from Fig. 1 above indicate that the majority (66 %) of clients received 2 or more psychotropic drugs from prescribers at a time. On the other hand, in about 34 % of data from prescription records, patients were given 1 drug after they were diagnosed.

**Fig. 2:**
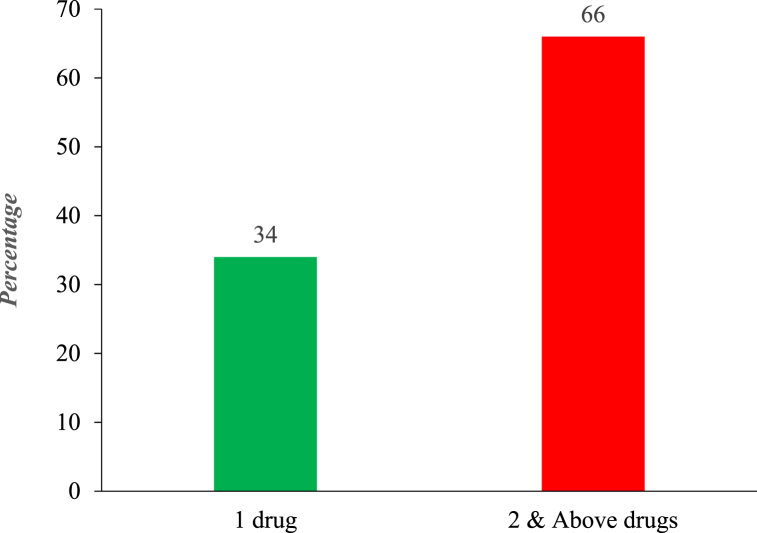
Prevalence of psychotropic polypharmacy (PP).

**Source**: **Fieldwork (2020).**

Aside from the PP prevalence above, the study as well also revealed the number of medications given to clients after diagnosis in various categories as indicated in the table below.

Results from Table 3 above indicate that about 105 clients out of 309 (representing 34 %) received 1 drug after diagnosis. Clients who took 2 psychotropic drugs were as well 72 representing 23.3 %. Also, clients who were prescribed 5 drugs were 19.4 %. Clients who took 6 or more psychotropic medications were the last group of only 1 %.

**Table 3:** Quantity of drugs received by clients.

Number of drugs	Freq. (N = 309)	Percentage (%)
1 drug	105	34.0
2 drugs	72	23.3
3 drugs	45	14.5
4 drugs	24	7.8
5 drugs	60	19.4
6 drugs & above	3	1.0

### 3.2 Category of psychotropic medications frequently prescribed

The study seeks to investigate the types or specific categories of psychotropic medications that are commonly prescribed to clients. These psychotropic medications that were given to clients in this study were therefore classified into various categories which are indicated in Table 4 below.

**Table 4:** Frequently prescribed psychotropic medications.

Drug categories	Freq. (N = 309)	Percentage (%)
Antipsychotics	230	74.4
Antidepressants	29	9.4
Mood stabilizers	18	5.8
Antianxiety	1	0.3
Anti-convulsant	31	10.0

Results from Table 4 of the study indicate that the majority (74.4 %) of the psychotropic medications that were frequently prescribed were mostly antipsychotic medications. Also, 10 % of the prescribed medications were anti-convulsant medications. In contrast, the least prescribed medication from the study was identified to be antianxiety medications which were only 0.3 % as shown above.

**Fig. 3:**
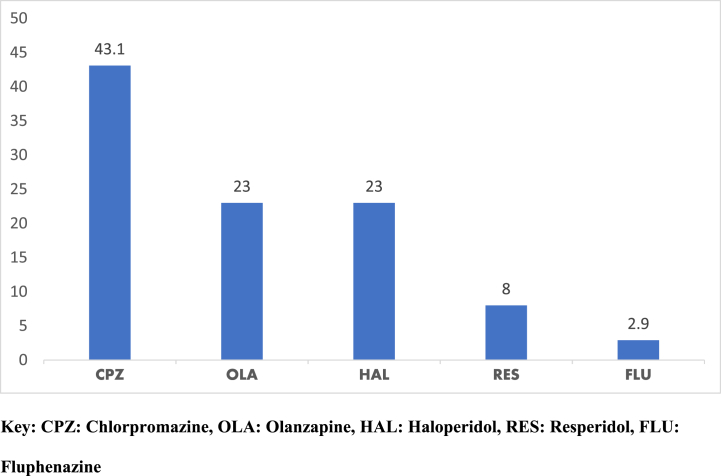
Antipsychotic prescription.

**Source**: **Fieldwork (2020).**

Moreover, the study also probed to ascertain which specific drug of the anti-psychotic class was frequently or mostly prescribed, findings on this are indicated in Fig. 3 below.

Results from Fig. 3 above clearly show that, close to half (43.1 %) of the antipsychotic medication commonly prescribed was chlorpromazine. Moreover, olanzapine was the next most frequent antipsychotic medication prescribed with 23 %. As indicated in Fig. 3 above, injection fluphenazine (2.9 %) was the least prescribed antipsychotic medication among them.

### 3.3 Concurrent combination of frequently prescribed antipsychotic medications

**Fig. 4:**
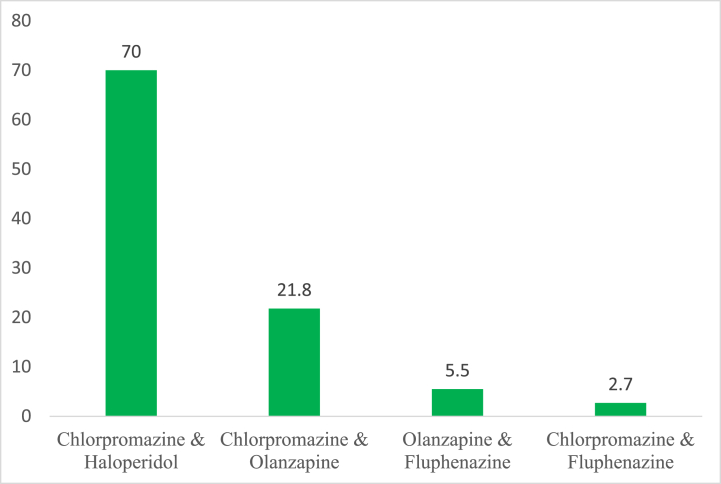
Combination of antipsychotic prescription.

**Source**: **Fieldwork (2020).**

The study also investigated how often prescribers (concurrent/simultaneously) or co-prescribed these anti-psychotic medications as per client per visit. The results from this analysis are indicated in Fig. 4 below.

Findings from this study revealed that, out of the frequently combined prescribed anti-psychotic medications, the majority (70 %) combined chlorpromazine & haloperidol to clients. Also, about 21.8 % of combined chlorpromazine & olanzapine to clients. However, the last combination was identified to be chlorpromazine & fluphenazine (2.7 %) as shown in Fig. 4 above.

It was also noted that, in most (about 80 %) of this combined prescription of antipsychotic medications, the tablet artane was simultaneously added to the clients.

### 3.4 Knowledge of prescribers (mental health workers) on psychotropic polypharmacy (PP)

The knowledge level (concerning PP meaning and its effects) of prescribers (mental health workers) was relevant to PP existence and therefore, this study examined their knowledge using the following variables in the table below.

Results from Table 5 indicate that more than half (65.5 %) of the respondents disagreed that prescribing only one drug to the client was PP. 22.4 % of them agreed that it was polypharmacy prescribing one drug to clients whilst only 3.5 % of the respondents were not able to tell if it was PP.

**Table 5:** Knowledge of prescribers on psychotropic polypharmacy.

Variable	Strongly Agree N (%)	Agree N (%)	Disagree N (%)	Not Sure N (%)
Meaning of psychotropic polypharmacy:				
Prescribe one drug at a time for patient	5 (8.6)	13 (22.4)	38 (65.5)	2 (3.5)
Prescribe two drugs from the same class at a time	23 (39.7)	21 (36.2)	13 (22.4)	1 (1.7)
Prescribe more than two from the same class for Patient	27 (46.6)	18 (31.0)	10 (17.2)	3 (5.2)
Using 2 or more psychotropic drugs for the same condition	25 (43.1)	19 (32.8)	13 (22.4)	1 (1.7)
***Effects of psychotropic polypharmacy:***				
Increase risk of liver and renal diseases	33 (56.9)	18 (31.0)	7 (12.1)	0
Cost to the patient and family	31 (53.5)	22 (37.9)	4 (6.9)	1 (1.7)
Poor or non-adherence to medication regimen	26 (44.8)	24 (41.4)	6 (10.3)	2 (3.5)
Adverse effects like drug reaction and interaction	20 (34.5)	28 (48.3)	9 (15.5)	1 (1.7)
Increased risk of drug toxicity	23 (39.7)	25 (43.1)	7 (12.1)	3 (5.2)
Risk of cognitive impairments	11 (19.0)	26 (44.8)	15 (25.9)	6 (10.3)
Potential relapse of the patient	12 (20.7)	30 (51.7)	11 (19.0)	5 (8.6)

Again, respondents were asked if prescribing two drugs from the same category was considered PP. Results from the study indicated that more than one-quarter of the prescribers (39.7 %) strongly agreed it was PP. On the contrary, 22.4 % of them disagreed with this as being PP and only 1.7 % of the prescribers were unable to tell if it was PP.

Prescribers were also asked if using two or more psychotropic drugs to treat the same condition equally constitutes PP. Findings from the study showed that close to half (43.1 %) strongly agreed, 22.4 %, disagreed with this, and 1.7 % were not sure if this could be PP.

With prescribers' knowledge relative to the effects of PP, the following were the respective responses to the statements made; on the extra cost to patients and families, about half (53.5 %) strongly agreed. Contrarily, 6.9 % of the respondents were also on the view that there is no cost, while 1.7 % were unable to tell if that was an extra cost to patients and families.

Prescribers again were asked if polypharmacy could have a risk of cognitive impairment in patients. The results above showed that close to 44.8 % agreed with this. On the contrary, 25.9 % of the prescriber disagreed with any risk of cognitive impairment to the patient. About 10.3 % were as well unable to tell if it could have this risk.

Moreover, 30 % of the respondents agreed that PP can contribute to patient relapse whilst 11 % disagreed.

### 3.5 Rating of prescribers' (mental health workers) knowledge of psychotropic polypharmacy

To determine prescribers’ knowledge level, questions that were measuring their knowledge based on responses were used to rate them. Respondents who scored 7 and above questions correctly were considered knowledgeable in PP. On the other hand, those who scored 6 and below correct were considered low knowledge of PP. This is indicated in Table 6 below.

**Table 6:** Rating of prescribers’ knowledge of psychotropic polypharmacy.

Knowledge Rate	Frequency	Percentage (%)
***High (7−11)***	48	82.8
***Low (0–6)***	10	17.2
***Total***	**58**	**100.0**

Findings from the study Table 6 indicated that a greater majority (82.8 %) of the prescribers scored between 7 and 11 correct, which indicated they had high knowledge of psychotropic polypharmacy. Contrarily, only 17.2 % of them scored between 0 and 6 indicating low knowledge of psychotropic polypharmacy.

### 3.6 Association between prescribers' (mental health workers) knowledge level and their demographic characteristics

The study sought to ascertain if respondents' (prescribers') respective knowledge levels were influenced by their characteristics. A Pearson chi-square correlational test was carried out to find out this relation and the results are shown in Table 7 below.

**Table 7:** Association between prescribers’ knowledge level and demographic characteristics.

Variable	Low Knowledge	High Knowledge	Chi-square/p-value
Gender			
Male	4 (11.1)	32 (88.9)	
Female	6 (27.3)	16 (72.7)	**2.449/0.114**
***Age groups***			
20–30yrs	7 (20.0)	28 (80.0)	
31–40yrs	2 (9.1)	20 (90.9)	6.011/0.050*
41–50yrs	1 (100)	0	
***Marital status***			
Married	4 (14.3)	24 (85.7)	
Single	6 (20.0)	24 (80.0)	**0.331/0.565**
***Qualifications***			
RMN	7 (19.4)	29 (80.6)	
CMHO	3 (14.3)	18 (85.7)	**0.459/0.795**
RGN	0	1 (100)	

Findings from Table 7, the chi-square test indicated that respondents’ gender, marital status, and qualification were statistically not significant with their knowledge level as indicated in the table above. However, respondents' ages were found to be statistically significant with their knowledge level (n = 58, ꭓ2 = 6.011, p = 0.050, α = 5 %).

### 3.7 Reasons prescribers (mental health workers) may consider PP as an option

This study as well seeks to investigate reasons why prescribers will consider PP as another option in treating their patients/clients. A series of questions were used to probe this as indicated in Table 8 below.

**Table 8:** Reasons for considering psychotropic polypharmacy.

Variable	Strongly Agree *N (%)*	Agree *N (%)*	Disagree *N (%)*	Not Sure *N (%)*
Prescribing more than one psychotropic to a patient will be considered or is acceptable in:				
The severity of the patient's condition.	23 (39.6)	20 (34.5)	13 (22.4)	2 (3.5)
Upon request or demand from the patient/family.	4 (6.9)	12 (20.7)	41 (70.7)	1 (1.7)
Availability of psychotropic medications.	3 (5.2)	11 (19.0)	44 (75.8	0
For speedy recovery of patients.	9 (15.5)	25 (43.1)	22 (37.9)	2 (3.5)
Cost/price of the psychotropic medication.	4 (6.9)	10 (17.2)	39 (67.2)	5 (8.6)

Findings from as in Table 8 of the study revealed that 39.6 % of prescribers strongly agreed that PP should be an option when the patient's condition is severe. On the contrary to this 22.4 % also disagreed with that fact, the severity of the condition should not lead to polypharmacy. However, 3.5 % were unable to tell if that could lead them to take that decision.

Again, respondents were asked if drug availability could usually influence their practice of PP at any time. The results indicate that the majority (75.8 %) disagreed with this as a pre-condition for PP practice. Only 19 % agreed that PP should be practiced under this circumstance.

Respondents were also asked if the speedy recovery of patients (expected desire) would influence the practice of PP. The results indicated that close to half (43.1 %) of the respondents confirmed that could happen. However, 37.9 % disagreed with the fact that speedy recovery does not or should not warrant PP and 3.5 % were not sure if this could be a factor.

### 3.8 Prescribers' view on the encouragement or promotion of psychotropic polypharmacy in psychiatry

Results in Table 9 which deals with the idea of encouraging psychotropic polypharmacy, more than half (68.9 %) of the respondents disagreed that PP should be encouraged or promoted in the treatment of patients. However, 19 % of the respondents supported that idea or statement whilst 6.9 % were unable to tell if it should be encouraged or not.

**Table 9:** Views of prescribers.

Variable	Freq. (N = 58)	Percent (%)
PP practice should be encouraged generally.		
Strongly Agree	3	5.2
Agree	11	19.0
Disagree	40	68.9
Not sure	4	6.9

### 3.9 Other findings from the study

**Fig. 5:**
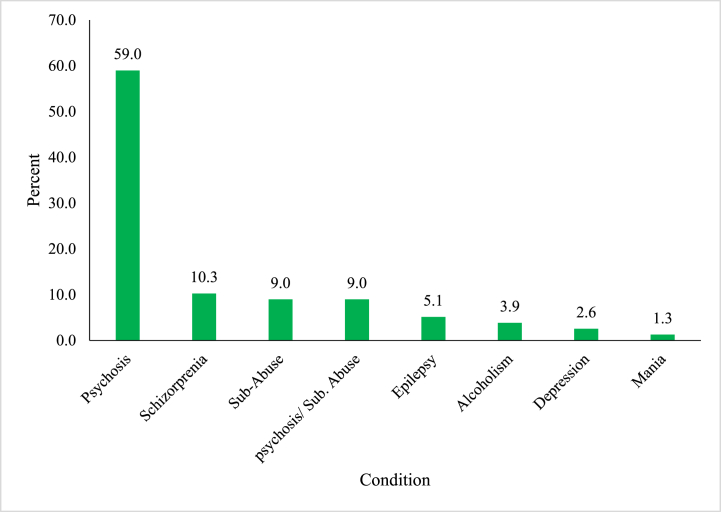
Prescription pattern relative to condition.

Source: Fieldwork (2020)

The study investigated respondents’ diagnoses concerning frequently prescribed psychotropic polypharmacy and came out with the following results as indicated in Fig. 5 below.

Results from the study show that the frequently occurring polypharmacy which is chlorpromazine & haloperidol was prescribed to most patients suffering from psychosis, followed by patients diagnosed with schizophrenia. On the other hand, only 1.3 % of patients diagnosed with mania were involved in PP.

### 3.10 Age and psychotropic polypharmacy

The study also found out the age group of patients that was more involved in PP and the findings are shown in the table below.

Results from Table 10 above show that more than one quarter (34.6 %) of the patients aged between 21 and 30 years were given the combination of chlorpromazine & haloperidol with about 1.3 % of patients who were 10 years and below taking this same combination. Again, the “chlorpromazine & olanzapine” combination was given to patients between the ages of 11–20years. The results also show that “chlorpromazine & fluphenazine” combined prescription to patients occurred mostly among respondents aged 21–30 years (66.7 %). About 50 % of the “olanzapine & fluphenazine” prescription was among the 31–40 years of age group.

**Table 10:** Age and psychotropic polypharmacy.

Age groups	CPZ & haloperidol	CPZ & Olan	CPZ & Fluphenazine	Olan & Fluphenazine
**10yrs & Below**	1 (1.3)	0	0	0
**11**–**20yrs**	8 (10.3)	***9 (37.5)***	0	0
**21**–**30yrs**	***27 (34.6)***	8 (33.3)	***2 (66.7)***	1 (16.7)
**31**–**40yrs**	19 (24.4)	4 (16.7)	0	***3 (50.0)***
**41**–**50yrs**	14 (17.9)	2 (8.3)	1 (33.3)	1 (16.7)
**51**–**60yrs**	6 (7.7)	1 (4.2)	0	1 (16.7)
**61yrs & Above**	3 (3.9)	0	0	0
**Total**	78 (100.0)	24 (100.0)	3 (100.0)	6 (100.0)

### 3.11 Sex and psychotropic polypharmacy

Furthermore, additional probing was done to find out which sex group of patients received these categories of polypharmacy and the table below shows the findings.

Results from Table 11 above showed that more than half (66.7 %) of the chlorpromazine & haloperidol combined prescription was among male patients with only 33.3 % among females. Also, the majority of the “CPA & olanzapine” polypharmacy occurred within male patients. However, more than half (66.7 %) of both chlorpromazine & fluphenazine” and “olanzapine & fluphenazine” polypharmacy occurred among female patients.

**Table 11:** Sex and psychotropic polypharmacy.

Drug combination	Male	Female	Total
CPZ & Haloperidol	52 (66.7)	26 (33.3)	78 (100.0)
CPZ & Olanzapine	18 (75.0)	6 (25.0)	24 (24.0)
CPZ & Fluphenazine	1 (33.3)	2 (66.7)	3 (100.0)
Olanzapine & Fluphenazine	2 (33.3)	4 (66.7)	6 (100.0)

## 4 Discussion

### 4.1 prevalence of psychotropic polypharmacy

A study in Nigeria reported a high prevalence of psychotropic polypharmacy (PP) ranging from 2.9 to 27 %, especially in children and adolescents [6]. In this study, the prevalence of psychotropic polypharmacy was assessed by identifying and categorizing clients according to the number of medications prescribed to them in each facility under study. The study found that only 34 % of clients who visited the facility were prescribed one drug while 66 % of clients were prescribed two or more psychotropic drugs. The breakdown of the 66 % of clients prescribed 2 or more drugs revealed that 23 % were prescribed exactly 2 drugs while 19 % were prescribed 5 drugs, 14 % were prescribed 3 drugs with 7.8 % and, 1 % prescribed 4 and 6 drugs respectively. This indicates that clients have a high probability of receiving more than two drugs on a visit to the facility.

The reasons for this prevalence of PP are due to lack or inadequate proper monitoring supervision of these prescribers by the authority and no regular training for prescribers to improve their skills or competences on the proper standards of prescription. Moreover, this could be attributed to the need and demand for fast treatment outcomes relative to the desire of prescribers and families of patients. Additional this can be said to prevalence due to inadequate proper review of cases at the OPD level. The perception and demand of patients for more drugs per visit in the Ghanaian health settings also sometime put undue pressure to prescribers to prescribe more drugs leading to PP.

The finding of this study corroborates with another study which found that the prevalence of polypharmacy in psychiatry varies between 13% and 90 % [8]. Similar studies in Europe also found that the prevalence of polypharmacy ranges between 33.3 % and 73.3. % depending on the adopted definition [9]. Another study of a cohort of elderly people in Bambuν city to evaluate the prevalence of polypharmacy and the influence of income on the association between medication use and cognitive impairment among elderly people [10]. They reported that within the geriatric age group (age >60 years), 44.8 % of the 1554 elderly Bambuν cohorts were consuming 2–4 medications and 25.5 % were consuming five or more medications.

The findings of this study also revealed that patients aged between 21 and 30 years were more (34.6 %) involved in PP and received chlorpromazine + haloperidol and chlorpromazine + fluphenazine combinations, but the combined prescription of olanzapine + fluphenazine was high (50 %) among patients aged between 31 and 40years. On the other hand patients, 61 years who were more likely to be diagnosed with dementia received less PP. This confirms the findings of another study that younger and unmarried patients were more likely to receive PP, hence exposing them to all the associated risks of PP [[11], [12]].

### 4.2 Category of psychotropic medications frequently prescribed in PP

A study using a nationwide representative sample of 3466 children and adolescents, reported the prevalence of multi-class psychotropic treatment to be 19 % in this population. Antidepressants were the most common co-prescribed medication class in multi-class visits followed by ADHD medications, antipsychotics, mood stabilizers, and sedative-hypnotics [13].

The study employed five categories to group the types of psychotropic drugs frequently prescribed; anti-psychotics, mood stabilizers, antidepressants, antianxiety, and anti-convulsant. Antipsychotic drugs were found as the most (74.4 %) prescribed category of medication, this corroborates the findings by another study which found that antipsychotic polypharmacy is widely prevalent, and all available evidence suggests that antipsychotic polypharmacy is common in clinical practice [14].

Anticonvulsant were 10 % of the prescribed category, anti-depressants were 9.4 %, with 5.8 % and 0.3 % for mood stabilizers and antianxiety respectively. These percentages indicate that clients are the least prescribed drugs from these categories in the facilities under this study. This finding is contrary to an earlier study which found that 6 % of patients received typical, while 39 % received atypical antipsychotics [15]. There was greater use of antidepressants (23 %), mood stabilizers (19 %), and antianxiety agents (16 %) relative to other studies. The use of anti-impulsive drugs, stimulants, and, hypnotics was rare (1–2 %).

It is however worth nothing that, unlike in Europe and Asia, the availability of psychotropic medications is mostly erratic in supply to mental health units. Most of the psychotropic medications available for use are largely the 1st generation antipsychotic medications unlike in the developed countries. Additionally, there exit affordability or cost issues or challenges which mostly influence prescriber choice.

A study found that half of the patients used in the study received combinations of non-clozapine oral antipsychotics and the other half were receiving either clozapine or an injectable antipsychotic [16]. The findings of this study found that the concurrent combination of frequently prescribed antipsychotic medications indicated that, the prescription of chlorpromazine with haloperidol was highest 70 %, chlorpromazine with olanzapine (21.8 %), olanzapine with fluphenazine (5.5 %), and chlorpromazine with fluphenazine 2.7 %. This finding in this study could support the general idea that most patients potentially experienced side effects of their medications including EPSE as the two most combined prescribed antipsychotic drugs are known and reported to have such potentials comparatively.

### 4.3 Knowledge of prescribers (mental health workers) in PP

Polymedicine or polytherapy is explained as the use of multiple medications prescribed appropriately for treating multiple comorbid conditions [17]. Polypharmacy is a simple term that can be said to be the prescription and use of many drugs at a time to manage a condition. The argument about polypharmacy is not solely about the number of medications used, but also about the effectiveness, utility, and potential harm of each medication, either individually or in combination [3]. The knowledge of the prescriber and pharmacy workers is very important because it influences the prescriptions given and pattern by a prescriber [18].

The study sampled 58 prescribers, 36 (62.1 %) are RMN, 21 (36.2 %) CMHO, and 1 (1.7 %) other health practitioners. In the assessment of the knowledge of prescribers on the meaning of polypharmacy, 65.5 % of prescribers disagreed that prescribing only one drug to the client was polypharmacy. 39.7 % of prescribers agreed the prescription of two drugs from the same category as polypharmacy, 22.4 % disagreed and 1.7 % were unable to tell. 43.1 % of prescribers indicated using 2 or more psychotropic medications to treat the same condition equally constitutes PP, with 22.4 % not in agreement and 1.7 % not sure if this could be PP. Prescribers’ knowledge relative to the effects of PP, about half (53.5 %) strongly agreed on the extra cost to patients and families. Contrarily, 6.9 % of the respondents were also on the view that there is no cost, while 1.7 % were unable to tell if that was an extra cost to patients and families.

When prescribers were asked if polypharmacy could have a risk of cognitive impairment in patients, close to 44.8 % agreed to this. On the contrary, 25.9 % of the prescriber disagreed with any risk of cognitive impairment to the patient. About 10.3 % were as well unable to tell if it could have this risk. Moreover, 30 % of the prescribers agreed that PP can contribute to patient relapse whilst 11 % disagreed. Therefore, prescriber had good understanding of PP and this could be attributed to the exposure during their training period and the type of health professional (clinical health workers).

A systematic study revealed that there were 111 numerical-only definitions of what polypharmacy means [17]. In all 80.4 % of the definitions, 15 quantity definitions that incorporated a duration of therapy or healthcare setting represented 10.9 % and 12 descriptive definitions as 8.7 %. The study also revealed that the most commonly reported definition of polypharmacy was the numerical definition of five or more medications daily (n = 51, 46.4 % of articles), with definitions ranging from two or more to 11 or more medicines. Only 6.4 % of articles were observed to have classified the variation between appropriate and inappropriate polypharmacy, which is using descriptive definitions to make this distinction. This study therefore focused on the multiple or numerical definition of PP which include both regular and prn prescriptions.

### 4.4 Rating of prescribers' (mental health workers) knowledge of PP

The lack of a consistent, clear, and globally accepted definition of polypharmacy including terms such as minor, moderate, and major polypharmacy, has made it pretty difficult for healthcare professionals to assess and consider efficacy and safety issues within the clinical setting [19]. A study indicated that most nurses do have the ability to assess the side effects of psychotropic medications, hence are capable of doing so relative to psychotropics poly-pharmacy [18].

Prescribers were rated to ascertain their overall knowledge of psychotropic polypharmacy based on their score in answering the 11 questions which were measured knowledge-based on responses. Respondents who scored 7 and above questions correctly were considered knowledgeable in PP and those who scored 6 and below correct were considered low knowledge on PP. It was found that 82 % were highly knowledgeable and 17.2 % had low knowledge in polypharmacy.

These findings however did not support the findings of the study which found that hospital pharmacists had low knowledge of psychotropic polypharmacy [18].

### 4.5 Association between prescribers' (mental health workers) knowledge level and their demographic characteristics

The study investigated the association between prescribers' (mental health workers) knowledge level and their demographic characteristics. Findings from the chi-square test indicate that respondents’ gender, marital status, and qualification were statistically not significant with their knowledge level. However, respondents' ages were found to be statistically significant with their knowledge level (n = 58, ꭓ2 = 6.011, p = 0.050, α = 5 %) which can be linked to their level of experiences gathered over the years on the field.

### 4.6 Reasons prescribers (mental health workers) may consider PP as an option

A study indicated that psychotropic polypharmacy is still a common practice despite inconclusive scientific evidence of additional efficacy and potential exacerbation of undesirable effects of medications [20]. A study on some reasons for polypharmacy practice stated antipsychotic polypharmacy (APP) might be appropriate in patients who have failed or cannot tolerate antipsychotic monotherapy put forward [13]. The safety data for psychotropic polypharmacy are mixed; several studies of clozapine plus risperidone or adjunctive aripiprazole suggest that these combinations generally are well tolerated with positive effects among patients.

Findings from this study revealed that 39.6 % of prescribers strongly agreed that PP should be an option when the patient's condition is severe whereas 22.4 % also disagreed severity of the condition should not lead to polypharmacy. However, 3.5 % were unable to tell if the severity of the condition could lead them to take that decision. This means that prescribers engaged in PP because they anticipated a particular effect(s) or treatment outcome, especially to the patients who reported to their units. This could also be because of psychotropic medications cost related or affordability and availability challenges in the country.

### 4.7 Other findings from the study

#### 4.7.1 Category of diagnosed patients and psychotropic polypharmacy

This study found out that, more patients (59 %) who were diagnosed with psychosis were involved in PP as compared to patients with Schizophrenia/Schizo-affective disorder of 10.3 %. These findings vary from that of an earlier study which indicated that patients diagnosed with Schizophrenia/Schizo-affective disorders were more likely to receive oral antipsychotics PP [21].

This variation could be attributed to probable misdiagnosing by these prescribers as they were likely not be make very specific or precise diagnosing as recommended by the DSM V, but rather lamping all psychotic features into the larger but undifferentiated or general psychosis diagnosis.

#### 4.7.2 Age and psychotropic polypharmacy

The study revealed that patients who were between the age group of 21–30 years received more (34.6 %) PP as compared to other age groupings. They received more combinations of chlorpromazine + haloperidol and 667 % of chlorpromazine + fluphenazine prescriptions. The co-prescription of olanzapine + fluphenazine was common among the 31–40years age group. However, patients above 61 years received less PP. This finding is consistent with the findings of a previous study which indicated that younger and unmarried patients suffering from psychiatric conditions were more likely to receive PP comparatively, therefore, exposing them to all the potential negative effects of PP like poor and decline/lower cognitive functioning including EPSE of the drugs [22].

### 4.8 Sex and psychotropic polypharmacy

In this study, it was revealed that male patients who visited the respective facilities were more (66.7 %) involved in PP with the combined prescription of chlorpromazine + haloperidol than females. However, in PP instances of combined prescription of chlorpromazine + fluphenazine, female patients were more dominant comparatively. These findings mean that male patients were at higher risk of experiencing EPSEs of drugs than female patients, for these combinations of drugs in PP is known to be among the high-risk drugs combination in PP as indicated by findings in a previous study [23].

## 5 Conclusions

The following major conclusions were drawn based on the main results of the study.

1. There is a high prevalence of PP in the study area and 2 or more psychotropic drugs were prescribed to a patient per visit (i.e., numerical PP). More patients diagnosed with psychosis were involved in PP and patients suffering from Mania were less involved in PP.
2. Prescribers within the study area had a high understanding of what PP was.
3. Same-class polypharmacy involving antipsychotic polypharmacy was the more common in the study area whilst antianxiety medications were least prescribed.
4. The combination of chlorpromazine and haloperidol (typical antipsychotic) was prevalent in the PP practice relative to antipsychotic polypharmacy and more males between the age group of 21–30 years received this combination than female patients.
5. The majority of the prescribers believed that PP in psychiatry should be discouraged but indicated that PP should be considered for the speedy recovery of the patient.

## Author contributions

Bernard Opoku Amoah: Writing – review & editing, Writing – original draft. Antwi Joseph Barimah: Writing – review & editing, Writing – original draft, Conceptualization. Yaw Boakye Nketiah: Writing – review & editing, Writing – original draft. Mohammed Mohammed Ibrahim: Methodology, Investigation. James Dumba: Writing – review & editing, Writing – original draft, Methodology, Conceptualization. Larry Agyemang: Writing – review & editing, Writing – original draft, Methodology. Frank Acheampong: Software, Formal analysis, Data curation. Luke Sopaal: Software, Formal analysis, Data curation. Deborah Ampofo: Writing – review & editing, Writing – original draft, Formal analysis. Rebecca Dorcas Commey: Writing – review & editing, Writing – original draft. Solomon Saka Allotey: Methodology, Investigation. William Appertey: Formal analysis, Data curation. Semefa Alorvi: Methodology, Investigation, Data curation

## Funding

No external funding was received for this research.

All authors contributed significantly to the critical revision and approved the final version before the onward submission.

## Declaration of competing interest

The authors declare that they have no known competing financial interests or personal relationships that could have appeared to influence the work reported in this paper.
